# Oocyte Cryopreservation at a Young Age Provides an Effective Strategy for Expanding Fertile Lifespan

**DOI:** 10.3389/frph.2021.704283

**Published:** 2021-09-22

**Authors:** Maurizio Poli, Antonio Capalbo

**Affiliations:** ^1^Igenomix Italy, Marostica, Italy; ^2^Centrum voor Kinderwens, Dijklander Hospital, Purmerend, Netherlands

**Keywords:** oocyte (egg) freezing, elective oocyte (egg) cryopreservation, social freezing, fertility preservation, age-related infertility, elective oocyte (egg) banking

## Abstract

With an upward trend in delaying parenthood, women across the world face an increasing risk of age-related infertility and involuntary childlessness. Elective oocyte banking strategies offer women the possibility to protect part of their reproductive potential until personal finances, personal relationship, or career have stabilized. Timely collection and cryopreservation of oocytes when they are most competent and chromosomal abnormality rates have not yet escalated are crucial for achieving high live births through *in vitro* fertilization (IVF) treatment at a later stage. To promote reproductive autonomy, women shall be informed about the decrease in fertility rates that sharply intensifies from the age of 35 years and the strategies available to maintain their reproductive potential. Together with this information, women should also recognize the limitations of available strategies including expected live birth rates, costs of the procedures, and overall approach performance, which is mainly associated with age at cryopreservation, number of oocytes banked, and age at accessing the banked oocytes. Evidence-based statistics are not yet available due to the relatively short period in which oocyte cryopreservation has been offered for elective purposes and the scarce number of patients returning for accessing their oocytes. However, to evaluate the applicability of fertility cryopreservation on a large scale, several theoretical models have been proposed to assess the expected efficacy and overall cost-effectiveness of different oocyte banking strategies. In this study, we review current oocyte cryopreservation methodologies, their applications, and outcomes. Moreover, we summarize current evidence regarding known parameters affecting oocyte banking efficacy. Finally, we discuss key points that could play a role in improving access to the service and optimization of oocyte banking frameworks.

## Introduction

Social and financial causes are driving a progressive and concerning trend of reproductive delay in most affluent countries. This is strongly shown by the increase in the average age of women carrying their first pregnancy, averaging at ~29 years in Organisation for Economic Co-operation and Development (OECD) countries (1). The causes for pregnancy postponement can be found, among others, in longer financial instability and a longer commitment to fostering the career of an individual before being able to “afford” to build a family (2). Moreover, a vast proportion of women cite the necessity of a stable long-term relationship as a key factor for considering pregnancy (3). However, the female reproductive potential is inversely correlated to age, and childbearing postponement exposes women to the risk of age-related infertility (4, 5). Involuntary childlessness becomes more frequent with the advancement of maternal age for which assisted reproduction is often sought as a remedy. Although *in vitro* fertilization (IVF) has been instrumental in the achievement of pregnancy for millions of couples in advanced reproductive age, it is well-demonstrated how successful IVF outcomes decrease with the increase in the age of the female patient (6). Low ovarian reserve, poor oocyte quality, and a fast-increasing rate of aneuploidy in oocytes after the age of 35 years limit the efficacy of assisted reproduction in mature patients (7). These restrictions could be circumvented using gametes sourced from young donors, which have proven to lead to high clinical outcomes in advanced maternal age patients, suggesting that ovarian ageing and its associated processes are the main cause for the decline in IVF outcomes for older patients (8). From a technical standpoint, current oocyte cryopreservation strategies produce extremely high survival rates and developmental potential of the ensuing embryo, providing confidence in the useability of cryopreserved gametes at a later stage (9). Initially specifically indicated for patients at greater risk of losing their fertility due to sterility-inducing medical intervention (i.e., oncological patients undergoing chemo/radiotherapy), the continuous improvement in clinical outcomes has widened the scope of oocyte cryopreservation applications. For example, oocyte cryopreservation is now mainly employed for banking gametes for oocyte donation programs. More recently, oocyte freezing has been employed in patients looking to extend their reproductive lifespan for more social reasons (i.e., delayed parenthood). According to recent estimations, procedures for elective oocyte cryopreservation are increasing faster in the UK, the US, and Spain and in developing countries (10). Although logically and scientifically sound, oocyte cryopreservation at a young age currently shows low cost-efficacy mainly due to the low rate of subsequent usage of the banked oocytes. In this study, we are summarizing current evidence on the use of oocyte cryopreservation as a strategy to extend fertility potential.

## Oocyte Cryopreservation

### The Development of Oocyte Cryopreservation Methodologies

The first human pregnancy from cryopreserved oocytes dates back to 1986 (11). At that time, oocytes were cryopreserved surrounded of the cumulus oophorus complex using the slow freezing method, which relied on introducing a membrane-permeable cryoprotectant [i.e., dimethylsulfoxide (DMSO)], and slowly cooling the specimen to around the freezing point (i.e., ~-7°C) using an automated cell freezer. Ice nucleation was then induced outside of the oocyte to drive the formation of ice crystals and prevent lethal damage of spontaneous ice origination within the cell. Since then, a wealth of research studies has been directed to the improvement of this methodology. Embryo cryopreservation was shown to produce better survival rates compared to oocytes, mainly due to the smaller water content of the cells and the increased surface-to-volume ratio of the former. However, ethical concerns and legal constrains in embryo cryopreservation in some countries (e.g., Italy) have boosted the need for more efficient oocyte cryopreservation protocols. Some of the improvements achieved in the early 00s involved the introduction of optimized cryopreservation media that included non-permeating cryoprotectant to further reduce the water content of the oocyte (e.g., sucrose and trehalose) and avoid spontaneous intracellular ice nucleation and the use of less toxic permeable cryoprotectants [e.g., ethylene glycol (EG) and propanediol (PrOH)]. During the last decade, slow freezing has been almost completely replaced by vitrification, a method that further minimizes the risk of the formation of ice crystals by lowering the freezing point of the sample (achieved by higher concentrations of cryoprotectant) and by dramatically increasing the cooling rate (12). This strategic combination delivers a crystal-free glass-like state cryopreserved specimen. With the optimization of vitrification, which included the reduction in loading volumes, the lowering of cryoprotectant concentrations and an increase in cooling rates, and oocyte survival and meiotic spindle re-polymerization rates improved significantly delivering higher outcomes in terms of clinical pregnancy rate per cycle compared to slow freezing strategy (13–15). One of the known effects of cryopreservation is zona hardening, which can be effectively overcome by employing intracytoplasmic sperm injection (ICSI), demonstrated by the high fertilization achieved (i.e., 70–80%). Storage duration does not appear to have a negative effect on oocyte post-warming viability (16). Reassuringly, gene expression of long-term cryopreserved gametes (6 years) was found similar to those stored for a shorter period (3 years), although transcriptomic profiles differed between cryopreserved and fresh oocytes (17).

### Applications of Oocyte Cryopreservation

Oocyte cryopreservation was initially practiced in those cases where the fertility of the patient was threatened by cancer, chronic illness, and/or the iatrogenic complications of treatments, where no other alternative was possible for preserving fertility (18). However, with the introduction of vitrification and the improvement in clinical outcomes, oocyte cryopreservation was extended to other contexts. For example, gamete cryopreservation is now indicated for patients diagnosed with benign conditions associated with early exhaustion of the reproductive potential (i.e., severe recurrent ovarian endometriosis, ovarian torsion, and familial history of premature ovarian insufficiency) (19). Unfortunately, late identification of such conditions may be associated with an already compromised prognosis, thus reducing the efficacy of the cryopreservation strategy. More notoriously, oocyte vitrification has found its main application in gamete donor programs where the possibility of desynchronizing the cycles of donor and recipient offered crucial advantages in treatment scheduling and oocyte availability, allowing for widespread implementation of donation programs based on international gamete cryobanks. More recently, oocyte cryopreservation has been employed for fertility preservation for so-called “social,” “non-medical,” or “elective” reasons (20). Essentially, these cases involve patients that choose to have a back-up of frozen oocytes in case later natural or IVF attempts with fresh oocytes don't work due to age-related infertility.

### Clinical Outcomes of Oocyte Cryopreservation

The effect of vitrification on fertilization and developmental potential has been investigated in several randomized clinical trials and prospective studies (21–24). In a study by Cobo et al., IVF outcomes deriving from fresh and vitrified sibling oocytes transferred to 300 matched recipients were compared, showing no significant difference in the ongoing clinical pregnancy rate (21). Similar findings were confirmed in other prospective studies where sibling oocytes allocated to vitrification or fresh donation programs showed no difference in fertilization, cleavage, embryo quality, and implantation rates (23–25). In a follow-up study, Cobo et al. monitored survival and clinical outcomes derived from the warming of over 40,000 donated oocytes. In this study, vitrification procedures delivered survival rates over 90% (26). Additional analysis showed that when employed in donation programs, oocyte vitrification can result in implantation, clinical pregnancy, and sustained clinical pregnancy rates as high as 39, 48, and 39%, respectively (26). Moreover, considering the same oocyte warming cycle, cumulative pregnancy rates (CPR) reached 78.8% after a maximum of five embryo transfers (26). However, a recent retrospective analysis of 1,844 oocyte donation cycles where 35,654 oocytes were split between fresh and frozen treatments showed inferior embryological and clinical outcomes for the vitrified oocytes (9). Nonetheless, after adjusting the data for the efficiency of warming, no differences between the groups were observed. This study demonstrated that when an equal number of useable oocytes are considered, fresh and frozen strategies deliver the same clinical outcome, highlighting how the performance of the cryopreservation program is crucial to obtain optimal results.

Furthermore, obstetrical and perinatal follow-ups of pregnancies originated from cryopreserved oocytes were conducted to assess the mid-term safety of the methodology. Cobo et al. observed no differences in obstetrics and perinatal outcomes from 2,251 newborns (1,027 and 1,224 from vitrified and fresh oocytes, respectively) in terms of obstetrical problems, gestational age at delivery, birth weight, Apgar scores of newborns, birth defects, admission to neonatal intensive care unit (ICU), and perinatal mortality (27). In addition, a review of 936 neonatal outcomes derived from cryopreserved oocytes showed no increased risk of congenital abnormalities compared to the incidence in the general population (1.3%) (28). Following the gathering of these reassuring results, oocyte cryopreservation was officially recognized as a clinical procedure and no longer an experimental methodology (29). Despite these reassuring results, the efficacy and safety of oocyte cryopreservation remain an important topic worth of careful monitoring. Prolonged follow-up of children born through oocyte vitrification should, therefore, be performed to ensure that no long-term effects are associated with this methodology.

## Elective Oocyte Banking

### Estimated Number of Oocytes Required to Obtain a Pregnancy

Estimating the chance of pregnancy in relation to age and number of oocytes cryopreserved is crucial for the counseling of patients interested in fertility cryopreservation and the creation of meaningful expectations for the treatment strategy. For this reason, several groups have undertaken analyses of embryological and clinical outcomes following oocyte cryopreservation. Rienzi et al. determined that delivery rates from vitrified oocytes were reproducible and consistent, although female age and the number of oocytes available influenced treatment outcome (30). Recursive partitioning analysis determined that clinical outcome was significantly improved when more than eight oocytes were available for treatment and that a reduced number of oocytes in patients older than 38 years of age dramatically reduced clinical outlook. Similar findings were observed by Doyle et al., where the threshold of 38 years of age determined better and worse outcomes with a sensitivity of 74% and specificity of 41%, and where patients under <38 years of age achieved a clinical pregnancy rate of 60.2%, while 43.9% was reached in patients >38 years of age (31).

In the same study, the efficiency of cryopreserved oocytes in generating a pregnancy was calculated based on cumulative results obtained from fresh and subsequent frozen embryo transfers in a population of 128 patients undergoing IVF treatment with autologous cryopreserved oocytes. Data showed that, with the advancement of age, more oocytes were required to achieve a live birth, with 7.4% efficiency in women that froze their oocytes before 30 years of age, 7.0% for women between 30 and 34 years of age, 6.5% for women between 45 and 47 years of age, and 5.2% for women over 40 years of age. Because of the small sample size, additional estimation tools were employed for calculating efficiency in groups of older women, resulting in an oocyte efficiency of 1.1% for patients aged 43–44 years (31). Overall live birth rate per warmed oocytes was calculated to be 6.5%.

In another study where age at freezing, estimated oocyte survival, blastocyst formation, and embryo euploidy rates per age group were considered in a theoretical model, patients cryopreserving 20 oocytes at the ages of 34, 37, and 42 years were expected to have a likelihood of 90, 75, and 37%, respectively, of achieving a live birth (32). Putting on another prospective, patients of similar age would require 10, 20, and 61 oocytes to achieve a 75% likelihood of live birth rate. If additional pregnancies were to be considered in the consultations of patients, the same age-groups with 20 cryopreserved oocytes may be expected to achieve a second live birth in 66, 39, and 7% of cases, and a third in 38, 15, and 1% of cases.

### Optimizing Timing for Elective Oocyte Cryopreservation

The age at which oocyte freezing is undertaken has important consequences both on the efficacy and the cost-effectiveness of the fertility preservation strategy. If oocytes are cryopreserved earlier, then a higher number of euploid gametes can be retrieved in fewer stimulation cycles, resulting in lower costs of the banking phase and higher reproductive potential. However, on the one hand, a young patient is statistically more likely to conceive naturally before the need of accessing the banked oocytes, resulting in lower cost-effectiveness of the procedure. On the other hand, an older patient would require more cycles of stimulation to reach a sufficient number of competent oocytes to provide a meaningful chance for a future pregnancy, thus raising the costs of the banking phase. In this case, though, due to the higher incidence of age-related infertility, the patient is less likely to conceive on her own, increasing the chances of using the banked oocytes and making her initial decision worthwhile.

In a study by Mesen et al., the theoretical chances of live birth were modeled based on the age at oocyte cryopreservation, personal requirements for oocyte utilization, and the time at which the patient would use the banked gametes. Here, a decisive factor determining the overall chances of live birth resulted to be whether the patient would use their banked oocytes (i) only if married or (ii) also in case of an unmarried partnership or using a sperm donor. Comparing the chances of live birth in the scenario of oocyte cryopreservation and no action, results were largely improved in the group where marriage was not a prerogative. In this group, the highest rates of live birth were reached when the patient banked her oocytes at a younger age. However, the oocyte cryopreservation strategy offered the biggest probability gain over no action when the patient cryopreserved her oocytes at 37 years of age with the plan to use them in 7 years of time (33). Shorter times between oocyte banking and utilization reduce the gain in chances of live birth produced by oocyte cryopreservation compared to no action. In all simulations where marriage was a necessary condition for utilization of the banked oocytes, the gain provided by cryopreservation over no action was never above 10%. Similarly, the previously mentioned study by Doyle et al. indicates that when freezing before 37 years of age, a rough estimate of 70–80% live birth rate can be achieved with 15–20 mature banked oocytes. When the patient is between 38 and 40 years of age, this estimate decreases to 65–70% starting from 25 to 30 mature banked oocytes (31).

### Cost-Effectiveness and Social Cost of Elective Oocyte Cryopreservation

Several studies have tried to determine the cost-effectiveness of elective oocyte cryopreservation. This task showed several limitations as multiple variables could not be input in the model as too complex or not properly characterized. Moreover, hypothetical assumptions and scenarios had to be made in order to build a theoretical model to predict the costs associated with fertility preservation through oocyte banking. These parameters, to mention some, included the age at which the patient freezes her oocytes, the time passed before returning to use them, fertility rates across ages, and the time allowed for spontaneous conception prior to embark either banked oocyte warming or IVF treatment. In a Europe-based study (where the cost of IVF is lower than the US and often subsidized by public welfare), Van Loendersloot et al. performed an analysis of the costs associated with a live birth in three different scenarios, where (i) the patient banked her oocytes at 35 years of age over 3 cycles of ovarian stimulation and returned for treatment at 40 years of age, (ii) the patient tried to conceive naturally between 40 and 45 years of age, and (iii) the patient tried to conceive naturally at 40 years of age and then moved to three cycles of IVF (34). In this model, fertility rates and IVF outcomes were based on the literature and institutional databases. Also, costs for each event (e.g., ovarian stimulation, oocyte freezing, IVF, gamete storage, embryo transfer, and miscarriage) were taken from national averages. According to this model, oocyte cryopreservation is a cost-effective strategy if 61% of patients with banked oocytes return to use their gametes and the cost of approximately $23,500 per birth is acceptable (as cost-effectiveness is subjective to the price willing to pay to achieve the desired result) (34). In another study, this time based on the US prices and policies, and Hirschfeld-Cytron et al. modeled costs and outcomes from oocyte cryopreservation at age of 25 years, no action, and ovarian tissue cryopreservation, with each strategy followed by four IVF rounds if unsuccessful (35). In this setting, ovarian tissue cryopreservation never showed higher cost-effectiveness compared to oocyte cryopreservation. Although oocyte cryopreservation is expected to produce a 7.5% increase in effectiveness compared to no action, it came with an additional cost per live birth of $135,000 (35).

From these diverging results, it is clear that, when calculating the cost-effectiveness of a strategy, a multitude of factors can influence theoretical models and their accuracy or adherence to reality. For example, the age at which the patient cryopreserves her oocytes (therefore, the associated statistical probability for her to return to use her oocytes) and the price estimates for each step of the treatment can significantly affect the cost-effectiveness calculation of live birth (36). Indeed, price points for interventions in the European study were lower than in the one from the US, highlighting how base costs do have a major effect on final cost-effectiveness.

In a study by Devine et al., three reproductive strategies were modeled, and sensitivity analysis was performed on fertility rates after 40 years of age and on the cost of both cryopreservation and fresh IVF treatment to identify how cost benefit per live birth changed in relation to these variables (37). The three strategies involved the following: (i) the patient cryopreserves a minimum of 16 mature oocytes at age 35 years with the intention to use them after the age of 40 years; when reaching 40 years of age, the patient tries natural conception for 6 months before returning to use the banked oocytes in two frozen IVF cycles; (ii) the patient banks oocytes at 35 years of age like in strategy (i), but after trying to conceive naturally for 6 months at the age of 40 years, she undergoes two rounds of fresh IVF before accessing the banked oocytes; and (iii) no oocyte cryopreservation undertaken by the patient which, after turning 40, tries natural conception for 6 months, followed by two rounds of fresh IVF if unsuccessful. Based on this model, strategy (i) was the most cost-effective with a mean cost per live birth of approximately $40,000 and 62% success in achieving live birth. Strategy (ii) resulted in the highest cost per live birth (~$62,000) but with the highest probability of success (74%). Strategy (iii) provided a 42% success rate with a cost per live birth of approximately $55,000. In this context, direct use of cryopreserved oocytes appeared to be always more cost-effective than undergoing fresh IVF prior to using banked gametes. Also, oocyte cryopreservation resulted in higher cost-effectiveness compared to fresh IVF at 40 years of age without banked oocytes if the oocytes were banked prior to reaching the age of 38 years. By increasing the theoretical chance of natural reproduction used in the model at the age of 40 years, the cost per live birth rapidly decreased for Strategy (iii). However, the cost-effectiveness of Strategy (i) remained superior until the natural fecundity rate was brought to 35%, which is two times higher than what detected in the general population (37). When modulating the cost for oocyte freezing, oocyte cryopreservation remained the most cost-effective strategy within the range of currently available costs. Oocyte cryopreservation combined with fresh IVF was more cost-effective than fresh IVF alone only when the cost of oocyte banking was below $9,300, which was a lower price point than the limit applied in this study. Similarly, Strategy (iii) showed better cost-effectiveness than the other approaches only when the cost of fresh IVF was modeled to be below the lower limit of current rates ($11,000). Similar to the previous studies, this study showed that, when patients plan to conceive at the age of 40 years, oocyte banking should be performed before the age of 38 years in order to reduce costs and increase success rates. Also, fresh IVF before using banked oocytes increases the chance of live birth, albeit reducing cost-effectiveness, while the use of banked oocytes alone produces more cost-effective live births (37). In this cost analysis, costs associated with miscarriage were not considered; however, as miscarriage would occur more frequently in patients undertaking Strategies (ii) and (iii) as they use 40 years old oocytes in either IVF or natural conception, their inclusion would have further improved the cost-effectiveness of oocyte banking. Miscarriage costs, as well as the further natural decline in ovarian physiology, would further increase the cost-effectiveness of oocyte banking, and reproductive attempts should be made after the age of 40 years.

Finally, while van Loendersloot analysis showed that oocyte cryopreservation was cost-effective if at least 61% of patients returned to access their gametes, the calculated threshold of Devine was at 49%. This discrepancy is probably due to the differences in the assumptions proposed to generate the statistical model.

### Limitations of Theoretical Predictive Models

The information provided by these studies, although theoretically modeled and, therefore, not fully accurate in a real scenario, could be extremely helpful in further counseling patients regarding the estimated number of cryopreserved oocytes required to achieve predetermined reproductive goals and to build more realistic expectations on the efficiency of the strategy considered. On the one hand, these figures also allow an estimation of the number of oocyte accumulation cycles required, improving the outlook on the predicted financial burden and expected return. On the other hand, theoretical studies may not be particularly accurate in determining the actual advantage produced by oocyte cryopreservation in a real context. For example, all these studies were conducted on data gathered from IVF clinics databases, which are populated by data from patients diagnosed with infertility and, therefore, a lower prognosis compared to the general population. Moreover, several assumptions were necessary in order to build a universally applicable theoretical model. For example, changes in performance across clinics (e.g., in terms of oocyte survival, blastocyst formation, and euploidy rates) severely affect the estimations provided by these models. A robust and comprehensive assessment of clinical performance based on actual clinical cases of patients returning to use cryopreserved oocytes is still missing, mainly due to small study population sizes as a result of patients not yet accessing their banked oocytes. Systematic reviews are being planned to define the efficacy of elective oocyte freezing in a real setting (38). These studies are expected to bring more information on the applicability of oocyte freezing for elective fertility preservation.

## Discussion

### Future Frameworks for Elective Oocyte Freezing

Oocyte cryopreservation for extending female reproductive lifespan can be seen as a preventive measure similar to “fertility insurance,” although no guarantees can be made about its success. Generally, the enrolment in this procedure isn't urgent, therefore it is crucial that comprehensive information on the rate of “protection”, costs, and realistic expectations is given to prospective patients to allow for fully informed decisions. However, as discussed above, the provision of such information currently requires assumptions and theoretical models that may not be representative of outcomes produced in the real context. For this reason, a better evaluation of the effectiveness of oocyte banking may be reached when a sufficient number of patients have undertaken it and adequate time has elapsed for them to require access to their gametes. In order to acquire this information in a timely manner, sharing of anonymized data across clinics and institutions would be advantageous. Meanwhile, additional policies may be investigated to promote and improve the accessibility and the projected cost-effectiveness of this service.

#### Awareness of Age-Related Fertility Decline and Reproductive Autonomy

Several studies have highlighted how a significant proportion of women are unaware of the age-related decline of fertility, often overestimating success rates achieved through IVF by advanced maternal age patients (39–41). Oocyte cryopreservation has caught the ear of the general public, mostly through the words of non-specialized magazines displaying a number of case reports of more and less famous women undergoing fertility preservation for non-medical reasons. These announcements have raised awareness about the availability of technologies enabling maternity postponement. However, proper information about the association between the outcomes and the timing and rate of fertility decline may still not be available to most. For example, the majority of patients undergoing oocyte cryopreservation indicate that if they had been aware, they would have proceeded earlier (42). Progressive campaigns on fertility should educate not only about when and how fast fertility declines in women but also about the limitations of current assisted reproduction technologies in fighting age-related infertility. Properly balanced pros and cons about when to cryopreserve gametes should also be provided to women of reproductive age, possibly through evidence-based data which, hopefully, will soon become available. As the direct association between the age at which a woman bears the first child and her academic education has been largely demonstrated (2, 43), public awareness programs may be more effective if first trialed in colleges and universities.

#### Cost Reduction for Oocyte Cryopreservation

In order to reduce the costs of oocyte cryopreservation processes, this service could be offered by clinics only specialized in fertility preservation, which, in cooperation with full IVF clinics, would later transfer the cryopreserved gametes to the IVF clinic chosen by the patient. Due to the reduced amount of technology employed in cryopreservation compared to full IVF, this strategy may optimize the price point of the service, reducing major overheads incurred by full IVF clinics, for which oocyte banking patients would not benefit. In addition, highly specialized clinics may be expected to achieve the best possible outcomes from cryopreservation techniques, further improving overall strategy efficiency. Moreover, when patients undergo ovarian stimulation at a younger age and without known fertility problems, a lower dosage of drugs may be required, thus lowering initial costs.

#### Identification of Patients at Higher Risk of Ovarian Depletion

Oocyte cryopreservation is already recommended for patients undergoing treatment for certain malignancies and non-oncological conditions. Some treatments have been associated with ovarian depletion, including hematopoietic stem cell transplantation for the treatment of several forms of anemia and alkylating agents for autoimmune conditions (44). Moreover, patients with ovarian endometriomas are subject to progressive ovarian depletion, further impacted when surgical treatment is employed (45). From a genetic standpoint, several disorders are associated with diminished and/or fast depletion of ovarian competence (e.g., Turner syndrome and galactosemia). Most of these conditions become apparent fairly early in life; hence, preventive actions to extend the fertility of patient can be timely adopted. However, other disorders affecting ovarian reserve may be asymptomatic and manifest only at a later stage when fertility potential is already compromised. Several tests have been developed for the assessment of ovarian reserve [e.g., early follicular phase **follicle**-stimulating hormone (FSH), estradiol and inhibin B, anti-Müllerian hormone (AMH), and antral follicle count], providing age-specific ranges that should ideally recognize early ovarian depletion. However, due to wide variations across individuals, these tests provide only partial sensitivity in discriminating the specific reproductive lifespan of the patient; hence, when suboptimal values are detected, ovarian competence is often already compromised, leaving patients with no options but to accept low reproductive prognosis (46). The use of family pedigree analysis and genome sequencing may improve the identification of patients requiring oocyte cryopreservation due to inheritable genetic traits associated with ovarian-related premature infertility. Although more evidence is required, an increasing number of genetic variants determining the predisposition to premature ovarian insufficiency and increasing oocyte aneuploidy rates are being identified through genome-wide association studies [reviewed by Capalbo et al. in 2021 (47)].

#### Framework for Donation of Unused Banked Oocytes

As discussed above, the rate of returning to access banked oocytes is a crucial parameter for determining the cost-effectiveness of elective oocyte cryopreservation. One way to improve this outlook could be offered by a framework that allows the release of the banked oocytes for heterologous donation or research studies. In several studies, when asked about these options, a variable portion of prospective patients were willing to donate their unwanted oocytes in case they did not need them (range 11-69%) (3, 48). However, this possibility would require the patient to comply with specific criteria (e.g., age at the time of cryopreservation, genetic and infectious disease screening, blood type, and Rh factor). Obviously, this approach has ethical implications that not every patient undergoing elective oocyte cryopreservation may be favorable to. Nonetheless, it may significantly improve the cost-effectiveness of the program.

## Conclusions

Oocyte cryopreservation is an effective strategy to mitigate the consequences of age-related infertility, offering high chances of parenthood to women that have to delay childbearing for non-medical reasons including the achievement of a fulfilling personal relationship, financial stability, or determining career goals. If undertaken sufficiently early in life, oocyte banking provides an extremely high probability to result in live birth and, therefore, has high efficacy in extending the fertile lifespan of a woman. Current evidence shows that, to achieve a 75% chance of live birth in subsequent cycles, oocyte cryopreservation should be undertaken before the age of 38 years, aiming at vitrifying between 10 and 20 mature oocytes, depending on the age of the patient at oocyte retrieval and the associated risk of chromosomal abnormalities. However, theoretical models calculating its cost-effectiveness show that live births achieved through oocyte banking may come at an increased social cost, especially if access and service optimization strategies are not followed. Operational cost reduction for oocyte freezing and potential rescue of “abandoned” cryopreserved oocytes remain underexplored options that may further improve the clinical, social, and economic outlook of this reproductive strategy. Nonetheless, large follow-up studies of patients undergoing elective oocyte cryopreservation are required to more accurately determine the efficacy and impact of this methodology based on clinical evidence.

## Author Contributions

MP researched, drafted, and reviewed the content of the manuscript. AC reviewed the manuscript. Both authors contributed to the article and approved the submitted version.

## Conflict of Interest

MP and AC are employees of Igenomix Italy, a genetic services provider.

## Publisher's Note

All claims expressed in this article are solely those of the authors and do not necessarily represent those of their affiliated organizations, or those of the publisher, the editors and the reviewers. Any product that may be evaluated in this article, or claim that may be made by its manufacturer, is not guaranteed or endorsed by the publisher.
